# Peptide Immunotherapy for Type 1 Diabetes—Clinical Advances

**DOI:** 10.3389/fimmu.2018.00392

**Published:** 2018-02-28

**Authors:** Emma L. Smith, Mark Peakman

**Affiliations:** ^1^UCB Pharma Ltd., Slough, United Kingdom; ^2^Department of Immunobiology, King’s College London, London, United Kingdom

**Keywords:** autoimmunity, type 1 diabetes, peptide immunotherapy, tolerance, antigen specific

## Abstract

Autoimmune and allergic diseases occur when an individual mounts an inappropriate immune response to a self-antigen or an innocuous environmental antigen. This triggers a pathogenic T-cell response resulting in damage to specific tissues and organs. In type 1 diabetes (T1D), this manifests as destruction of the insulin-secreting β cells, resulting in a life-long dependency on recombinant insulin. Modulation of the pathogenic T-cell response with antigen-specific peptide immunotherapy offers the potential to restore the immune homeostasis and prevent further tissue destruction. Recent clinical advances with peptide therapy approaches in both T1D and other diseases are beginning to show encouraging results. New technologies targeting the peptides to specific cell types are also moving from pre-clinical development to the clinic. While many challenges remain in clinical development, not least selection of the optimal dose and dosing frequency, this is clearly becoming a very active field of drug development.

## Introduction

Type 1 diabetes (T1D) is an organ-specific autoimmune disease. Auto-reactive T cells attack the insulin-producing beta (β) cells of the pancreatic islets, leading to a loss of endogenous insulin production and subsequent impaired glucose metabolism. Management of the disease requires daily administration of exogenous insulin and frequent monitoring of blood glucose levels. While there have been significant advances in recombinant human insulins and technologies both to deliver insulin and monitor blood glucose levels, many patients do not achieve optimal glycemic control. This is particularly apparent in children and young adults, in whom blood glucose control, measured by glycated hemoglobin (HbA1c) levels, is typically poor, reaching greater than 8% in the majority when the desired levels are below 7.5% (1). The longer-term complications associated with elevated levels of HbA1c are significant and include retinopathy, nephropathy, and neuropathy. Clearly, there is a need for new therapies that either preserve or restore β-cell function to improve glycemic control and patient outcomes.

Preservation of an individual’s own endogenous insulin secretion by modulation of the immune system would be the optimal solution. However, the major challenge for this therapeutic approach is that at the time of diagnosis, a considerable loss of β cells has already occurred (2). Encouragingly, there are now supportive data to suggest that preservation of residual β-cell function at the time of diagnosis may indeed lead to improvements in glycemic control (3, 4) and thereby impact upon long-term outcomes. Preventing immune-mediated attack on β cells would also open the possibility for β-cell regeneration and/or replacement therapies to be more effective and durable.

## Peptide Immunotherapy

**Figure 1:**
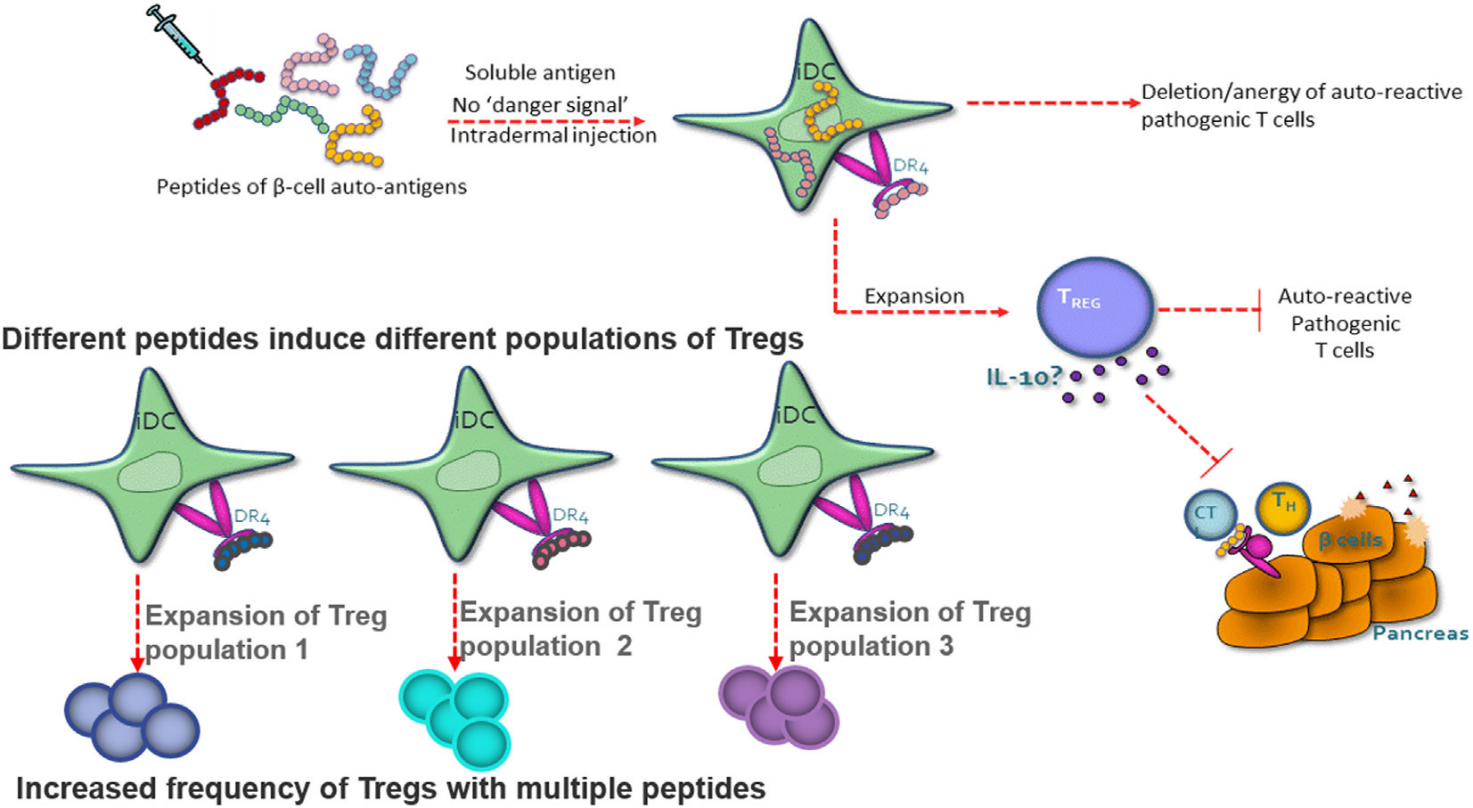
Peptide therapy restores immune homeostasis *via* natural antigen-specific immunoregulatory pathways. Highly soluble peptides are taken up by immature dendritic cells and presented to antigen-specific CD4^+^ T cells. The CD4^+^ T cells have one of the three potential fates; death, anergy, or the expansion/generation of a regulatory T-cell phenotype. The regulatory T cells suppress the pathogenic T cell *via* IL-10 secretion. Each peptide has the capacity to induce a different population of regulatory T cells.

Autoimmune and allergic diseases arise when an individual mounts an inappropriate immunological response to a self-antigen or an innocuous environmental antigen (5). Antigen-reactive T cells become activated and expand in number, and the regulatory T-cell pool is no longer capable of controlling the immune response, leading to the loss of immune homeostasis. Peptide immunotherapy offers the potential to restore immune homeostasis *via* the expansion of the regulatory T-cell pool and the deletion and/or anergy of the pathogenic T-cell population (Figure 1).

Clinical proof of concept that peptide therapy can restore immune homeostasis has been championed by studies of allergic disease. Cat-PAD is a peptide cocktail consisting of seven peptides from Feld1, the major cat allergen. A recent clinical study has shown that a short course of intra-dermal treatment with these peptides resulted in a clinically meaningful reduction in rhino-conjunctivitis symptoms, 2 years post-treatment (6). Disappointingly, a large phase III study failed to confirm the earlier positive clinical data (ClinicalTrials.gov: NCT01620762). This may in part be explained by a large placebo effect that was observed and the transition to field-based studies, which inherently introduce greater variability. An ongoing clinical study focusing on the phenotype of the allergen-specific T cells following administration of Cat-PAD will confirm whether peptide therapy can modulate the pathogenic T-cell response (ClinicalTrials.gov: NCT02311413).

Clinical studies with peptides derived from auto-antigens for the treatment of autoimmune disease are also now emerging. A phase IIa study with a peptide immunotherapy treatment for relapsing remitting multiple sclerosis demonstrated statistically significant reductions in total and new T1 Gadolinium enhancing brain lesions (representing sites of inflammation and damage) measured using magnetic resonance imaging during treatment. This therapy consists of a cocktail of four peptides derived from myelin basic protein.^1^ Two recent phase I studies with a peptide immunotherapy treatment for celiac disease have also shown encouraging data suggesting that the antigen-specific T-cell responses can be modulated following peptide treatment (7). Further development of NexVax2, a mix of three peptides derived from gluten, is planned.

## Peptide Immunotherapy for T1D

For peptide therapy to be successful in treating autoimmune or allergic disease, it is essential that the major auto-antigens responsible for driving the disease are known. Using the presence of auto-antibodies to the same antigens as a guide increases the probability that appropriate antigens have been selected. The ability to detect antigen-specific T cells is also important not only to aid the initial epitope discovery of disease-relevant peptides but also to track modulation of the T-cell phenotype following clinical administration.

**Figure 2:**
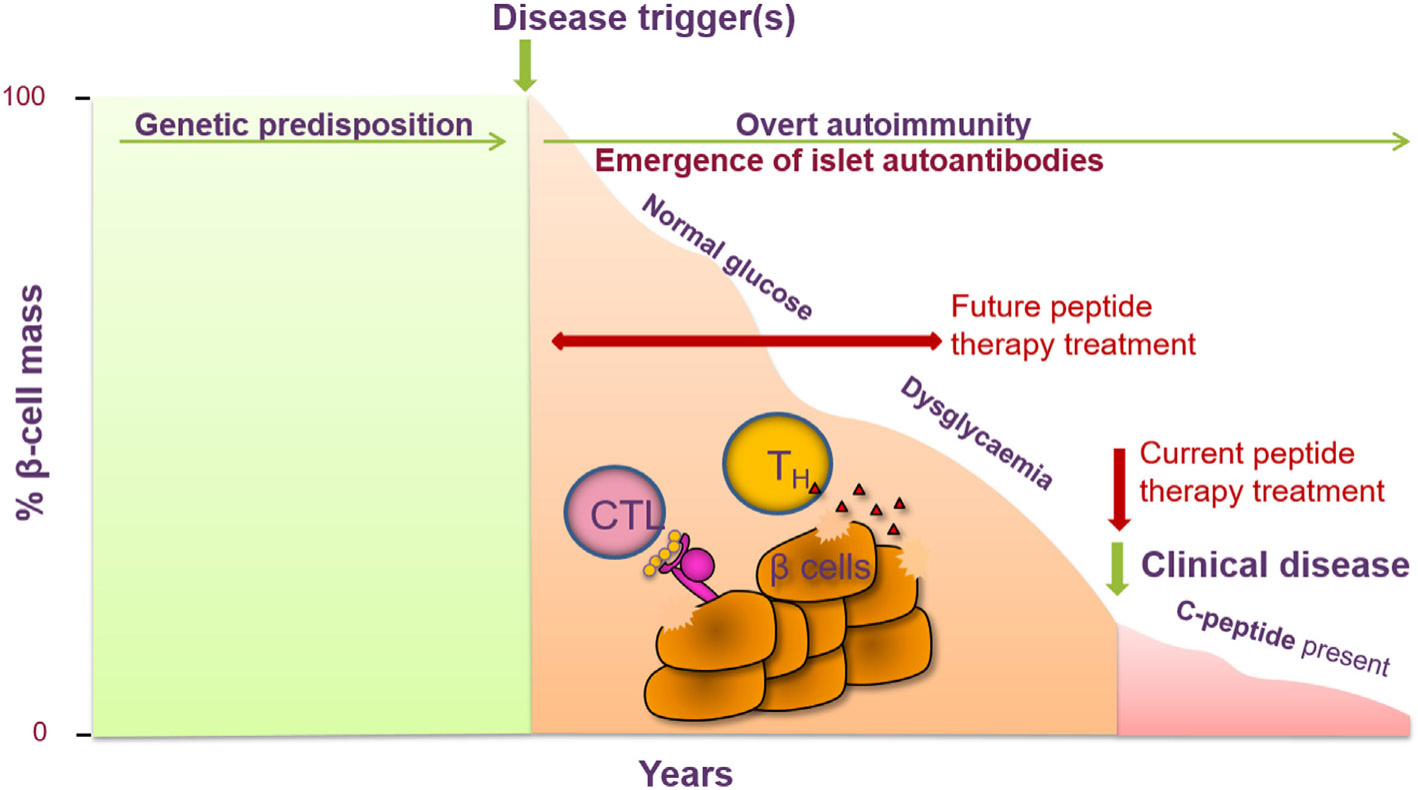
Peptide therapy for type 1 diabetes (T1D) offers the opportunity to halt further progression of disease in newly diagnosed individuals and to prevent clinical symptoms of disease in at-risk individuals. T1D is a T-cell-mediated autoimmune disease characterized by the destruction of the pancreatic β cells, resulting in a decrease in insulin secretion (measured *via* c-peptide). Genetic factors have been identified as contributing to the risk of developing T1D as well as certain environmental factors. Prior to the clinical symptoms of T1D, auto-antibodies to one or more islet cell antibodies are detected and individuals become dysglycaemic. Current intervention with peptide therapy is focused on treating newly diagnosed patients. Future invention studies aim to treat individuals prior to clinical diagnosis in the prevention setting.

Type 1 diabetes emerges as an ideal autoimmune disease for trialing treatment with peptide therapy. A number of the major antigens have been identified, including proinsulin, GAD65, IA-2, and ZnT8. Auto-antibodies to these antigens can be detected both in the sera of patients at the time of diagnosis and in individuals who are at high risk of future disease, whether they are entirely asymptomatic with no signs of disease, or have early indications of impaired glucose tolerance (8). Antigen-specific T cells from the blood of patients and at-risk subjects can be detected and, indeed, exhibit a proinflammatory cytokine response upon antigen stimulation *ex vivo* (9, 10). It is appealing to propose that progression of disease toward diabetes in high-risk subjects will be more tractable to therapies such as peptide administration than the scenario of waiting for the disease to become established. In the future, this will need to be tested in multiple antibody-positive non-diabetic subjects, whose risk of disease development over a lifetime approaches 100% (Figure 2).

An early clinical study in T1D patients administered an altered peptide ligand (APL) derived from the insulin B9-23 epitope (NBI-6024) showed some initial promise (11). Modulation of the antigen-specific T-cell response from a predominantly IFNγ response to a more Th2 bias was detected. However, a subsequent clinical study powered to assess the effect of repeated administrations of NBI-6024 on endogenous insulin production, as measured by C-peptide levels in adult and adolescent patients with new-onset T1D, failed to demonstrate any clinical benefit (12).

In parallel to the clinical development of NBI-6024, a natural peptide sequence derived from proinsulin was also under evaluation. An initial phase 1 safety study in patients with long-standing diabetes and low c-peptide levels (<200 pmol/L) compared three monthly intra-dermal injections of 10 versus 100 µg of the C19-A3 peptide (13). The peptide was shown to be well tolerated and safe. In addition, as was observed in the initial NBI-6024 study, there was a trend toward modulation of the antigen-specific T-cell response with an apparently increased frequency of IL-10 producing cells in the low-dose treatment group.

Subsequently, a second clinical study with the single proinsulin peptide was conducted in newly diagnosed patients and the results have recently been reported (14). In this study, monthly versus fortnightly intra-dermal injections of 10 µg of peptide were compared, over a period of 6 months. The peptide was shown to be well tolerated and safe. Although the study was not powered to examine efficacy at the level of preservation of β-cell function, there was evidence in some patients on the active treatment that the rate of decline in secreted c-peptide slowed and daily insulin use and HbA1c stabilized. Intriguingly, these subjects who could be labeled “treated c-peptide responders” also showed immunological changes compared with non-responders on active treatment, including greater propensity for CD4 T-cell production of IL-10 upon *ex vivo* stimulation and enhanced expression of the canonical regulatory T cell transcription factor FoxP3 on peripherally generated, adaptive regulatory T cells post-therapy. These findings, linking clinical and immunological outcome provide an encouraging platform on which to build strategies for biomarkers of peptide immunotherapy.

The clinical studies described thus far, both in T1D and the other indications, have focused on either a single peptide to modulate the pathogenic T cell response or multiple peptides from the same antigen. A different approach, using multiple peptides from two different auto-antigens linked to the pathogenesis of T1D, is currently being evaluated as a treatment for new-onset T1D patients (ClinicalTrials.gov: NCT02620332). This phase Ib tolerability and safety study evaluating monthly intra-dermal injections of a cocktail of multiple peptides should provide new insights when data become available in 2018.

A slightly different peptide therapy approach to those previously described is also under clinical evaluation. The approach is to take a natural peptide derived from one of the major auto-antigens linked to T1D and modify it slightly to improve its affinity (15). These modified peptides are known as Imotopes™. Injection of the Imotopes™ is believed to elicit antigen-specific cytolytic CD4 T cells that induce lysis of antigen-presenting cells (APCs) with which a synapse is formed, as well as the auto-antigen-specific bystander T cells, activated on the surface of the same APC. A phase Ib safety and tolerability study is currently underway, exploring three different doses administered sub-cutaneously in alum, with study completion expected December 2018 (ClinicalTrials.gov: NCT03272269).

## Promising Pre-Clinical Peptide Therapy Approaches

The peptide therapy approaches leading the way in clinical development for both T1D and other autoimmune diseases are relatively simple. The peptides are administered either intra-dermally or sub-cutaneously in the absence of any additional substantial modifications. Whether these approaches are sufficient to bring significant clinical benefit in large phase III clinical studies remains to be seen, but certainly, the promise peptide therapy holds for restoration of immune regulation has led a number of pharmaceutical companies to develop novel and innovative ways to target the peptides in a more specific manner for potentially enhanced efficacy. These include targeting of peptides to the erythrocyte (16), encapsulating peptides into nanoparticles (17–19) and encapsulating peptides into liposomes with α-GalCer.^2^ A highly novel approach being developed involves coating peptide-MHC complexes onto nanoparticles to generate “navacims” (20). As yet, there are no data to indicate which method of peptide delivery will be optimal and bring the greatest clinical benefit, but clearly this is a fast evolving field.

## Future Challenges and Opportunities

While peptide therapy for T1D is clearly entering an exciting phase of drug development, there are still a number of unanswered questions and challenges, not least whether the approach will bring real clinical benefit. Selecting the optimal dose of peptide and optimal dosing interval is one key challenge. This is in part because it is difficult to study both the kinetics of peptide uptake and the bio-distribution of peptides. Access to better immunological tools to track the antigen-specific T cells will undoubtedly help our understanding; this includes further development of both tetramers and *ex vivo* assays that selectively stimulate antigen-specific cells (21). The ability to sample T cells at the site of active disease and/or site of peptide administration may also provide greater insight into the mechanism of action of peptide therapy. It is generally believed that peptide therapy induces a population of IL-10 secreting regulatory T cells. However, this is primarily based on *in vivo* data from mouse studies that have clearly demonstrated expansion of an IL-10 secreting, regulatory T-cell population following sub-cutaneous administration of peptide (22). Translation of this mechanism into the clinical setting is still to be proven and may well depend on the route of administration of the peptides. Nonetheless, as discussed earlier, circulating IL-10 producing, antigen-specific CD4 T cells have been observed post-intra-dermal peptide administration in studies in T1D patients and this mode of action remains a major focus of attention.

Once the technologies and assays have been further refined so that tracking of antigen-specific T cells, both pathogenic and regulatory, can be accomplished, additional questions can start to be addressed. Importantly, degrees of clinical efficacy can begin to be correlated with both the frequency of regulatory T-cell expansion and the phenotype of the expanded regulatory T cells. Is better efficacy achieved *via* the generation of IL-10 producing regulatory T cells, the Foxp3+ regulatory T cell, or a combination of both? It will also be important to understand the longevity of the effect and whether this is dependent on the phenotype of the regulatory T cell. It may be that the different routes of administration of peptides and the newly emerging targeted technologies mentioned previously lead to the generation of different populations of regulatory T cells. It is not inconceivable to think that different diseases may be better treated by induction of one particular population of regulatory T cell versus another. In addition, different stages of progression within the same disease may be more amenable to treatment by one subset of regulatory T cell over another. This may be particularly true for diseases where there is significant epitope spreading. In this situation, one might envisage early in disease using a peptide treatment that induces the Foxp3+ regulatory T cell and later in the disease pathogenesis, using a peptide treatment that induces the IL-10 secreting regulatory T cell.

One must also acknowledge that administration of peptide therapy alone may not be sufficient to prevent disease in an inflammatory setting. In this setting, the peptide therapy may indeed be inducing a population of regulatory T cells, but the inflammatory environment may also be driving a pathogenic T-cell population, which is not adequately suppressed by the regulatory cells. Therapies that directly inhibit the pathogenic T cell while sparing the regulatory T cell would be optimal under these circumstances. These could include therapies that target the APC to prevent full activation, achieved for example using antibodies against CD40. Alternatively, therapies that inhibit effector T-cell function directly could be beneficial. This could be achieved either *via* inhibition of T-cell co-stimulatory receptors such as CD40L, OX40, and ICOS or *via* engagement of negative regulators such as PD-1 and TIGIT. Modulation of the cytokine milieu may also synergize with the efficacy of peptide therapy. This could be achieved *via* direct inhibition of the proinflammatory cytokines secreted by the pathogenic T cell or inhibition of cytokines responsible for maturation of the APC.

While the challenges are undoubtedly there, peptide therapy does offer a significant opportunity to restore immune regulation in T1D and other autoimmune diseases. Further clinical studies and technological advances will hopefully translate peptide therapy into a safe, effective, and highly specific novel class of therapeutics.

## Author Contributions

ES wrote the review and was involved in designing the figures. MP was involved in designing the figures and supervised the writing and editing of the manuscript.

## Conflict of Interest Statement

ES is an employee of UCB Pharma Ltd. who are developing peptide immunotherapy for type 1 diabetes. MP is employed by King’s College London, which has a licence agreement in place with UCB Pharma to develop peptide immunotherapy. MP receives research funds and honoraria from UCB.

## Footnotes

^1^https://apitope.com/apitope-announces-positive-atx-ms-1467-phase-iia-data-relapsing-multiple-sclerosis/

^2^http://www.regimmune.com/product-pipeline/tid/
